# Brain amyloid burden, sleep, and 24-hour rest/activity rhythms: screening findings from the Anti-Amyloid Treatment in Asymptomatic Alzheimer’s and Longitudinal Evaluation of Amyloid Risk and Neurodegeneration Studies

**DOI:** 10.1093/sleepadvances/zpab015

**Published:** 2021-09-19

**Authors:** Adam P Spira, Vadim Zipunnikov, Rema Raman, Jiyoon Choi, Junrui Di, Jiawei Bai, Cynthia M Carlsson, Jacobo E Mintzer, Gad A Marshall, Anton P Porsteinsson, Roy Yaari, Sarah K Wanigatunga, John Kim, Mark N Wu, Paul S Aisen, Reisa A Sperling, Paul B Rosenberg

**Affiliations:** 1 Johns Hopkins Bloomberg School of Public Health, Baltimore, MD, USA; 2 Johns Hopkins University School of Medicine, Baltimore, MD, USA; 3 Johns Hopkins Center on Aging and Health, Baltimore, MD, USA; 4 Alzheimer’s Therapeutic Research Institute, University of Southern California, San Diego, CA, USA; 5 Wisconsin Alzheimer’s Disease Research Center, University of Wisconsin School of Medicine and Public Health, Madison, WI, USA; 6 Ralph H. Johnson VA Medical Center, Charleston, SC, USA; 7 Lowcountry Center for Veterans Research, South Carolina Institute for Brain Health, Charleston, SC, USA; 8 Center for Alzheimer Research and Treatment, Brigham and Women’s Hospital, Massachusetts General Hospital, Harvard Medical School, Boston, MA, USA; 9 University of Rochester School of Medicine and Dentistry, Rochester, NY, USA; 10 Eli Lilly and Company, Indianapolis, IN, USA

**Keywords:** Alzheimer’s disease, amyloid, sleep, circadian, actigraphy, cognitive, rhythm, activity, wrist

## Abstract

**Study Objectives:**

To examine in a subsample at the screening phase of a clinical trial of a β-amyloid (Aβ) antibody whether disturbed sleep and altered 24-hour rest/activity rhythms (RARs) may serve as markers of preclinical Alzheimer’s disease (AD).

**Methods:**

Overall, 26 Aβ-positive (Aβ+) and 33 Aβ-negative (Aβ−) cognitively unimpaired participants (mean age = 71.3 ± 4.6 years, 59% women) from the Anti-Amyloid Treatment in Asymptomatic Alzheimer’s (A4) and the Longitudinal Evaluation of Amyloid Risk and Neurodegeneration (LEARN) studies, respectively, wore actigraphs for 5.66 ± 0.88 24-hour periods. We computed standard sleep parameters, standard RAR metrics (mean estimating statistic of rhythm, amplitude, acrophase, interdaily stability, intradaily variability, relative amplitude), and performed a novel RAR analysis (function-on-scalar regression [FOSR]).

**Results:**

We were unable to detect any differences between Aβ+ and Aβ− participants in standard sleep parameters or RAR metrics with our sample size. When we used novel FOSR methods, however, Aβ+ participants had lower activity levels than Aβ− participants in the late night through early morning (11:30 pm to 3:00 am), and higher levels in the early morning (4:30 am to 8:30 am) and from midday through late afternoon (12:30 pm to 5:30 pm; all *p* < .05). Aβ+ participants also had higher variability in activity across days from 9:30 pm to 1:00 am and 4:30 am to 8:30 am, and lower variability from 2:30 am to 3:30 am (all *p* < .05).

**Conclusions:**

Although we found no association of preclinical AD with standard actigraphic sleep or RAR metrics, a novel data-driven analytic method identified temporally “local” RAR alterations in preclinical AD.

**Statement of Significance**

Disturbed sleep and altered 24-hour rest/activity rhythms (RARs) are linked to cognitive decline, but few studies have examined associations of objectively measured sleep and RARs to Alzheimer’s disease biomarkers. We investigated whether cognitively unimpaired older adults who were β-amyloid (Aβ)-positive on positron emission tomography scans differed from those who were Aβ-negative on actigraphic measures of sleep and both conventional and novel, data-driven RAR metrics. Although we found no differences in conventional sleep or RAR indices between Aβ-positive and Aβ-negative participants, novel function-on-scalar regression analyses revealed differences between groups in both mean 24-hour activity patterns and their variability. Therefore, cognitively unimpaired older adults with and without Aβ deposition may exhibit differences in 24-hour RARs that may not be detected by standard circadian parameters.

## Introduction

Disturbed sleep and altered circadian rhythms are increasingly studied as potential risk factors for the development of Alzheimer’s disease (AD). Although sleep disturbance was once viewed solely as a consequence of AD pathology, animal studies have demonstrated that this is too simplistic a perspective. Sleep deprivation has been shown to increase β-amyloid (Aβ) deposition in AD mouse and *Drosophila* models [1, 2], providing evidence for a possible causal role of sleep loss in the AD pathological process. On the other hand, in an AD mouse model, the sleep/wake cycle has been observed to deteriorate as Aβ aggregates, suggesting that sleep disturbances can also serve as a marker of Aβ deposition [3]. Consequently, the association between disturbed sleep/wake behavior and Aβ deposition is considered to be bi-directional, with poor sleep leading to Aβ aggregation, which itself disturbs sleep, which promotes Aβ deposition, etc. [4]

The animal studies of sleep and Aβ are paralleled by a growing number of observational studies in humans. Among community-dwelling older adults, reports of poorer sleep quality and shorter sleep duration have been linked to greater Aβ deposition, measured by [^11^C] Pittsburgh compound B (PiB) positron emission tomography (PET) [5]. Compared to those who were Aβ-negative (Aβ−), cognitively unimpaired older adults who were Aβ-positive (Aβ+; measured by cerebrospinal fluid [CSF] Aβ) consistent with preclinical AD, had poorer sleep efficiency (i.e. spent a smaller proportion of time in bed asleep) and greater wake after sleep onset (WASO), measured by wrist actigraphy [6], an objective sleep-assessment method using accelerometers to quantify sleep and wake [7]. Recent work has also tied altered actigraphic 24-hour rest/activity rhythms (RARs) to Aβ burden in preclinical AD. In cognitively unimpaired older adults, Musiek et al. demonstrated that, compared to Aβ− participants, those who were Aβ+ on PiB PET had greater within-day RAR fragmentation, yet greater day-to-day RAR stability and greater amplitude, measured by actigraphy [8]. These findings complement prior investigations that have identified substantial 24-hour RAR disruption among older adults with clinical dementia [9].

Because sleep and circadian rhythm alterations may promote or result from AD pathology, they may serve as modifiable risk factors for, or markers of AD pathology [4, 10]. To better understand these associations, we collected wrist actigraphy data from a subset of cognitively unimpaired older adults who were Aβ+ in the ongoing Anti-Amyloid Treatment in Asymptomatic Alzheimer’s (A4) Study, a randomized controlled trial of the Aβ monoclonal antibody, solanezumab, and from a subset of cognitively unimpaired older adults in the Longitudinal Evaluation of Amyloid Risk and Neurodegeneration (LEARN) cohort, which consists of participants who were screened for the A4 Study and found to be Aβ− [11, 12]. Here, we present cross-sectional results from the screening phase of both cohorts, comparing actigraphic sleep and 24-hour RAR indices. Standard cosinor and nonparametric RAR metrics are single summary values that may obscure between-group differences that are restricted to narrower intervals in the 24-hour day. Thus, in addition to calculating standard 24-hour RAR metrics, we applied a data-driven nonparametric method, function-on-scalar regression (FOSR) [13], that may be more sensitive than conventional metrics to such time-limited differences. We hypothesized that the presence of Aβ would be associated with shorter sleep, greater wakefulness after sleep onset, and longer sleep onset latency (SOL), and both weaker and phase-delayed RARs.

## Methods

### Participants

As part of an actigraphy substudy, we studied 26 Aβ+ cognitively unimpaired older adult participants from the A4 Study and 33 Aβ− cognitively unimpaired older adult participants from the LEARN cohort. Participants were drawn from five A4/LEARN sites (Johns Hopkins School of Medicine, Baltimore, MD; University of Wisconsin School of Medicine and Public Health, Madison, WI; Roper St. Francis Hospital, Charleston, SC; Brigham and Women’s Hospital, Boston, MA; and University of Rochester School of Medicine and Dentistry, Rochester, NY). At screening, participants were included if they were ages 65 to 85 years old, had a Mini-Mental State Examination (MMSE) [14] score of 25 to 30, a global Clinical Dementia Rating (CDR) [15] score at screening of 0 (“normal”), and a Wechsler Logical Memory II [16] delayed recall score at screening of 6 to 18. These inclusion criteria were intended to recruit participants who were cognitively and functionally unimpaired. A4 participants had Aβ+ status on florbetapir PET scans. A4 used a quantitative standard uptake value ratio (SUVr) threshold of ≥1.15 to define “elevated amyloid” (Aβ+), instead of requiring positivity on a visual read. An SUVr from 1.10 to 1.15 was considered elevated amyloid only if a two-reader consensus determination was also considered positive [12]. The LEARN cohort consists of participants who are Aβ− but otherwise, meet all A4 eligibility criteria (i.e. A4 screen fails based on Aβ status). After completing PET scans and enrolling in A4 or LEARN, participants were invited to join the actigraphy substudy. Participants were excluded from enrolling in this substudy if they had a known medical condition that could affect the validity of actigraphy or inferences that might be drawn from it (e.g. severe tremor, obstructive sleep apnea, rapid eye movement sleep behavior disorder, restless leg syndrome) or used medications for sleep (i.e. benzodiazepines at night, trazodone, non-benzodiazepine hypnotics, tricyclic antidepressants, or melatonin or diphenhydramine for sleep). Institutional Review Boards from each study site approved the study protocol prior to performance of any study-related procedures.

### Wrist actigraphy

Participants were asked to complete seven 24-hour periods of wrist actigraphy with the Actiwatch-2 actigraph (Philips Respironics, Bend, OR) worn on the nondominant wrist with data collected in 30-second epochs. Participants were asked to press an event-marker button on the device once each night when they got into bed with the intention of sleeping (i.e. “lights out”), and once in the morning when they got out of bed to begin the day. Button presses produced time stamps in the data. Participants were also asked to complete a sleep log once each morning, to record “lights out” time the night before, time out of bed that morning, and the timing of any naps, device removals, or unusual sleeping circumstances (e.g. sleeping away from home, shift work, emergencies) or time-zone crossings. We used data from the event-marker time stamps, sleep diaries, and ambient light levels to identify the period that was most likely to be the in-bed interval and applied a validated algorithm [17] to generate sleep parameters using Actiware software (Philips Respironics). Two trained scorers worked independently to identify the in-bed interval and reviewed any discrepancies in their scoring. If they could not agree, a third trained scorer was asked to help resolve the issue. We studied the following sleep parameters: total sleep time (TST; time spent asleep while in bed); WASO (time spent awake after sleep onset); average wake bout length (average duration of wake bouts); sleep efficiency (% time in bed spent asleep); SOL (interval from time into bed to sleep onset).

To quantify 24-hour RARs, we used the following preprocessing steps. First, 30-second activity counts (AC) were log(AC + 1)-transformed to make their distribution more symmetric. Supplementary Figures 1 and 2 demonstrate the effect of this transformation on raw ACs and the distributions of raw and transformed ACs from one Aβ+ participant (Supplementary Figure 1A–D) and one Aβ− participant (Supplementary Figure 2A–D). Then, 30-minute binning was performed by averaging log-transformed AC within 48 nonoverlapping 30-minute intervals. Finally, the mean subject-specific RARs were obtained by averaging daily curves over all valid days. Thus, in the main analysis, each mean subject-specific RAR has been represented via 48 30-minute log-transformed ACs. We also derived subject-specific SD 24-hour RAR profiles by calculating the SD for each 30-minute epoch across all valid days. Finally, in a sensitivity analysis, we repeated these steps to create hour-level mean and SD RARs using 60-minute binning (more details provided below).

We calculated standard one-component cosinor RAR metrics, including MESOR (mean estimating statistic of rhythm; average activity level); amplitude (peak-to-trough difference; rhythm strength); and acrophase (timing of the rhythm’s peak) [7] using the “cosinor” package (https://cran.r-project.org/web/packages/cosinor/cosinor.pdf) in R software [18]. As an example, Supplementary Figure 3 shows average RAR profiles and fitted standard one-component cosinor models for one Aβ+ participant (Supplementary Figure 3A) and one Aβ− participant (Supplementary Figure 3B). Because RARs do not necessarily adhere to a cosinor shape, we also computed standard nonparametric statistics, including interdaily stability (IS; consistency of rhythm across days); intradaily variability (IV; rhythm fragmentation); and relative amplitude (RA; a rhythm-strength metric) [9, 19] using the “ActCR” package in R (https://cran.r-project.org/web/packages/ActCR/index.html). Further, we applied FOSR, a nonparametric data-driven method using the “fosr()” function from the “refund” package in R (https://cran.r-project.org/web/packages/refund).

### Other measures

Participants provided demographic data (i.e. age, sex, race, educational attainment) at screening. They completed several cognitive tests comprising the Preclinical Alzheimer Cognitive Composite (PACC) [20], including the MMSE [14], Logical Memory Delayed Recall [16], Free and Cued Selective Reminding Task Total Recall Score [21], and Digit Symbol from the Wechsler Adult Intelligence Scale – Revised (WAIS-R) [22]. The CDR [15] was also administered to a study partner and the participant. Apolipoprotein E (ApoE) genotype was obtained and participants were categorized by ApoE ε4 status (ε4+ vs. ε4−) as described by Sperling et al. [12]

### Statistical analyses

We computed descriptive statistics for Aβ+ and Aβ− participants and compared them using two-sample *t-*tests for continuous variables and Fisher’s exact tests for categorical variables. Subject-level sleep parameters were averaged over all available valid nights. To examine differences between Aβ+ and Aβ− participants’ sleep parameters and standard parametric and nonparametric RAR indices, after accounting for potential confounders, we also performed linear regression analyses with Aβ status as the predictor and sleep or RAR metrics as the outcome, adjusting for age, sex, and educational attainment. In addition to standard RAR metrics, we used FOSR. FOSR is a flexible nonparametric data-driven technique that assesses the time-varying associations between (mean or SD) subject-specific RAR profiles and subject-specific clinical characteristics and demographics [13, 23]. Specifically, adjusting for age, sex, and educational attainment, the FOSR model is as follows:


$$\begin{array}{ll} \left(1\right)\;RAR_{i}\left(t\right)= & \beta_{0}\left(t\right)+age_{i}*\beta_{1}\left(t\right)+\\ & sex_{i}*\beta_{2}\left(t\right)+education_{i}*\beta_{3}\left(t\right)+\\ & A\beta_{i}*\beta_{4}\left(t\right),\;t=1,\ldots ,\;\;\;48 \end{array}$$

where subindex i indicates subject. FOSR model (1) estimates the adjusted relationship between the functional outcome, RAR_*i*_(*t*), and the variable of interest, Aβ status, at each 30-minute epoch t. Assuming smoothness of functional outcomes, RAR_*i*_(*t*), FOSR enforces similar smoothness on functional regression parameters for the functional intercept, β _0_(*t*), the functional effect of age, β _1_(*t*), the functional effect of sex, β _2_(*t*), the functional effect of education, β _3_(*t*), and the functional effect of Aβ status, β _4_(*t*). Thus, FOSR could be viewed as an extension of the standard scalar linear regression with a flexibility of having a time-varying association between the outcome and the predictor of interest, described by functional or time-varying regression parameter β _4_(*t*). Eight basis functions were used and the penalized generalized least square method was chosen for estimation and selection of the tuning parameters in FOSR model. To test statistical significance, we used three increasingly conservative inferential methods: (1) pointwise confidence intervals; (2) simultaneous confidence intervals; and (3) a global *F*-test [24, 25]. All methods assumed the significance level of α = 0.05. Simultaneous confidence intervals attempt to take into account the information about the proximity of the adjacent time epoch and always deliver more conservative results compared to pointwise confidence intervals. Finally, although for completeness, we use and report the results based on the global *F*-test recently proposed in the functional data analysis literature, global hypothesis testing in a functional regression framework is not very well established, so the results based on the *F*-test should be interpreted cautiously, especially in applications with relatively small sample sizes [25]. All analyses were conducted using the statistical software R (www.r-project.org). All testing was two-sided and a *p*-value of .05 was considered statistically significant.

### Sensitivity analysis

Our main analysis described above uses mean and SD subject-specific RARs represented via 48 30-minute log-transformed ACs. To guarantee numerical stability of the FOSR model, we limited the number of epochs, 48, to be lower than the number of the subjects, 59. This choice allows us to keep a relatively high temporal resolution of 30-minute epochs which, in turn, increases a chance of identifying temporally local associations between mean and SD RARs and Aβ status. However, historically, parametric and nonparametric RAR analysis has been performed using ACs based on 60-minute epochs. Thus, in the sensitivity analysis, we re-ran all the tests and FOSR models using mean and SD RARs based on 60-minute epochs.

## Results

There were no significant differences (*p* < .05) between the 4,486 participants in the overall A4/LEARN screening PET sample and the 59 participants in the present substudy with respect to any of the participants characteristics described in Table 1, except for florbetapir SUVr, in which substudy participants had higher values (1.17 ± 0.22 vs. 1.09 ± 0.19, *p* = .009). There was a statistical trend toward a higher proportion of ApoE ε4 carriers among the substudy participants than in the overall A4/LEARN samples (47% vs. 35%, *p* = .072).

**Table 1.** Participant characteristics, mean ± SD or *n* (%).

	All participants	Aβ+ (A4)	Aβ− (LEARN)	P
	*N* = 59*	*n* = 26	*n* = 33	
Age	71.27 ± 4.63	72.41 ± 4.57	70.38 ± 4.55	.095
Female	35 (59)	17 (65)	18 (55)	.436
Apolipoprotein E ε4 carrier	27 (47)	16 (62)	11 (34)	.063
Non-Hispanic White	59 (100)	26 (100)	33 (100)	—
Education (years)	16.56 ± 2.38	16.00 ± 2.38	17.00 ± 2.32	.111
Mini-Mental State Examination	28.66 ± 1.25	28.50 ± 1.27	28.79 ± 1.24	.388
Free and Cued Selective Reminding Task	75.53 ± 6.04	74.42 ± 6.64	76.39 ± 5.47	.228
Logical Memory Delayed Recall	11.63 ± 3.30	11.31 ± 3.36	11.88 ± 3.28	.515
Digit Symbol	42.02 ± 9.23	43.73 ± 9.20	40.67 ± 9.17	.209
Clinical Dementia Rating, Sum of Boxes	0.04 ± 0.14	0.06 ± 0.16	0.03 ± 0.12	.646
Florbetapir standard uptake value ratio (SUVr)	1.17 ± 0.22	1.38 ± 0.13	1.00 ± 0.08	<.0001

*N = 58 for apolipoprotein E ε4 status.

Substudy participants had a mean ± SD age of 71.27 ± 4.63 years and 16.56 ± 2.38 years of education (Table 1). Overall, 59% were female and all were non-Hispanic White, 73% were married, 83% retired, 98% lived in their own home, and 69% had a family history of dementia. There were no statistically significant demographic, cognitive, or functional differences between Aβ+ (A4) and Aβ− (LEARN) participants. Compared to Aβ− (LEARN) participants, Aβ+ (A4) participants (by definition) had a higher florbetapir SUVr (1.38 ± 0.13 vs. 1.00 ± 0.08, *p* < .0001). In the whole sample (both groups combined), the median interval between PET scans and actigraphic assessment was 52 days (interquartile range [IQR]: 32–121). In the A4 (Aβ+) group it was 28.5 days (IQR: 21–38.5), and in the LEARN (Aβ−) group it was 93 days (IQR: 62–308).

### Sleep parameters and standard 24-hour RAR metrics

Participants wore actigraphs for 5.66 ± 0.88 24-hour periods (range 2 to 6). They obtained an average of 421.17 ± 52.01 min of TST, 37.76 ± 14.81 min of WASO, average wake bout length of 1.32 ± 0.30 min, 87.00 ± 5.34% sleep efficiency, and SOL of 11.81 ± 14.58 min (Table 2). There were no differences between Aβ+ versus Aβ− participants with respect to these sleep parameters in unadjusted pairwise comparisons or multivariable linear models adjusted for age, sex, and education (Table 3).

**Table 2.** Actigraphic sleep parameters, mean ± SD

	All participants	Aβ+ (A4)	Aβ− (LEARN)	*P*
	*N* = 59	*n* = 26	*n* = 33	
Total sleep time (min)	421.17 ± 52.01	416.82 ± 49.99	424.60 ± 54.07	.570
Wake after sleep onset (min)	37.76 ± 14.81	38.40 ± 15.46	37.25 ± 14.50	.772
Average wake bout length (min)	1.32 ± 0.30	1.32 ± 0.31	1.32 ± 0.30	.977
Sleep efficiency (%)	87.00 ± 5.34	87.14 ± 4.72	86.89 ± 5.86	.855
Sleep onset latency (min.)	11.81 ± 14.58	10.77 ± 9.79	12.63 ± 17.58	.608

**Table 3.** Adjusted* associations of Aβ status with actigraphic sleep parameters

	*B* (standard error) *P*				
	Total sleep time (min)	Wake after sleep onset (min)	Average wake bout length (min)	Sleep efficiency (%)	Sleep onset latency (min)
Aβ+ (A4) *n* = 26	−10.81 (13.92) *P* = .441	0.31 (4.17) *P* = .941	−0.04 (0.08) *P* = .590	−0.03 (1.49) *P* = .983	−0.06 (3.94) *P* = .988
Aβ− (LEARN) *n* = 33	(ref.)	(ref.)	(ref.)	(ref.)	(ref.)

*Adjusted for age, sex, education.

Two participants had fewer than three valid days of 24-hour epoch-by-epoch actigraphy data and were therefore excluded from 24-hour RAR analyses. With regard to standard cosinor RAR metrics measured on the 30-minute scale, participants’ mean amplitude was 2.30 ± 0.35, MESOR was 3.16 ± 0.32, and acrophase was 14:43 pm ± 1:04 am (Table 4). In terms of nonparametric RAR metrics, their mean RA was 0.81 ± 0.07, IS was 0.75 ± 0.08, and IV was 0.41 ± 0.13. There were no differences between Aβ+ and Aβ− participants in standard cosinor or nonparametric indices on the 30-minute scale in either unadjusted pairwise comparisons, or multivariable-adjusted analyses (Table 5). Nor were there differences when using data on the 60-minute scale (Supplementary Tables 1 and 2).

**Table 4.** Standard 24-hour rest/activity rhythm (RAR) indices (30-minute intervals), mean ± SD

	All participants	Aβ+ (A4)	Aβ− (LEARN)	P
	*N* = 57	*n* = 26	*n* = 31	
Cosinor indices				
Amplitude	2.30 ± 0.35	2.34 ± 0.33	2.28 ± 0.37	.516
MESOR	3.16 ± 0.32	3.20 ± 0.28	3.12 ± 0.35	.354
Acrophase	14:43 pm ± 1:04	14:32 pm ± 0:45	14:53 pm ± 1:15	.195
Nonparametric indices				
Relative amplitude (RA)	0.81 ± 0.07	0.81 ± 0.06	0.81 ± 0.08	.780
Interdaily stability (IS)	0.75 ± 0.08	0.75 ± 0.07	0.76 ± 0.08	.451
Intradaily variability (IV)	0.41 ± 0.13	0.40 ± 0.10	0.42 ± 0.15	.620

**Table 5.** Adjusted* associations of Aβ status with standard 24-hour rest/activity rhythm (RAR) indices (30-minute intervals)

				*B* (standard error) *P*		
	Cosinor indices			Nonparametric indices		
	Amplitude	Mesor	Acrophase	RA	IS	IV
Aβ+ (A4) *n* = 26	0.07 (0.09) *P* = .468	0.11 (0.09) *P* = .229	−0.35 (0.30) *P* = .249	0.01 (0.02) *P* = .710	−0.02 (0.02) *P* = .370	−0.02 (0.04) *P* = .494
Aβ− (LEARN) *n* = 31	(ref.)	(ref.)	(ref.)	(ref.)	(ref.)	(ref.)

IS, interdaily stability; IV, intradaily variability; RA, relative amplitude.

*Adjusted for age, sex, education.

### Exploratory RAR analyses

**Figure 1.**
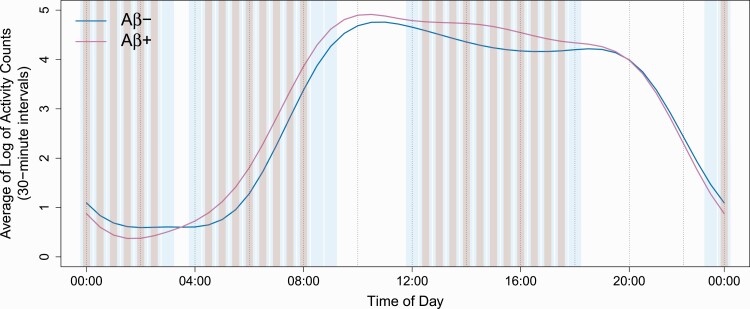
Between-group differences in mean activity profiles (30-minute intervals). When we used simultaneous (more conservative) confidence intervals to evaluate statistical significance, Aβ+ (A4) participants had lower mean activity levels (mean log activity counts) across days compared to Aβ− (LEARN) participants between 11:30 pm and 3:00 am and higher levels between 4:30 am and 8:30 am and between 12:30 pm and 5:30 pm (pink bars identify intervals with significant differences, *p* < .05). More extensive statistically significant differences emerged when the less conservative pointwise method was applied to generate confidence intervals (blue bars).

Some statistically significant differences emerged by Aβ status when we applied the data-driven FOSR models using 30-minute interval data. When nominal significance was determined based on the more conservative, simultaneous method of generating confidence intervals, Aβ+ participants had lower mean log activity levels than Aβ− participants from 11:30 pm to 3:00 am, and higher activity from 4:30 am to 8:30 am and from 12:30 pm to 5:30 pm (*p* < .05, Figure 1). When we used the less conservative, pointwise method of generating confidence intervals, nominally significant between-groups differences were evident across wider temporal intervals. When we applied a global *F*-test, examining whether on average Aβ+ and Aβ− participants exhibited distinct levels of activity across the entire 24-hour day, the results were nonsignificant (*p* = .409).

**Figure 2.**
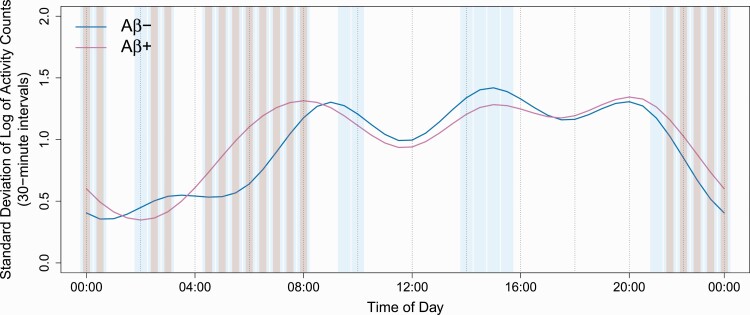
Between-group differences in variability (SD) of activity profiles (30-minute intervals). Across days, Aβ+ (A4) participants have greater variability in activity (SD of log activity counts) than Aβ− (LEARN) participants from 9:30 pm to 1:00 am and from 4:30 am to 8:30 am, and lower variability from 2:30 am to 3:30 am (*p* < .05) (pink bars identify intervals with significant differences, *p* < .05). When the less conservative pointwise method was used to test significance (blue bars), the intervals containing differences expanded slightly, and new regions of difference appeared in the mid-morning and early to midafternoon.

When we used simultaneous confidence intervals to investigate variability in RARs across days, Aβ+ participants had a higher SD of log AC than Aβ− participants, indicating greater variability in activity levels across days, between 9:30 pm and 1:00 am and from 4:30 am to 8:30 am (*p* < .05, Figure 2). Conversely, Aβ+ participants had lower variability (greater stability) in activity levels between 2:30 am and 3:30 am (*p* < .05). As with mean activity levels, we observed broader time intervals with nominally significant effects when we examined diurnal variability using the less conservative pointwise method, as well as intervals from 9:30 am to 11:00 am and from 2:30 pm to 3:30 pm in which Aβ+ participants had lower variability in activity levels across days. As shown in Figure 1, the difference in average RARs between Aβ+ and Aβ− participants can be up to 0.5 units on the log-scale. Combining this with the range for SD RARs shown in Figure 2, we see that this difference in average RARs can be translated into a half of SD, which is a noteworthy effect. When we used a global *F*-test to measure between-group differences in diurnal variability of activity across 24-hour intervals, however, results did not reach significance (*p* = .063).

In sensitivity analyses, we repeated the FOSR analyses using 60-minute epoch RARs. Substantially fewer significant between-group differences emerged when we analyzed the data at this coarser level of temporal resolution. When we used the more conservative simultaneous confidence interval method of establishing nominal statistical significance, compared to Aβ− participants, those who were Aβ+ had lower mean levels of activity from 2:00 am to 3:00 am (Supplementary Figure 4). Two additional intervals with significant differences were evident when we used the pointwise method to generate confidence intervals. We observed a similar pattern for SD, or variability of activity profiles (Supplementary Figure 5).

## Discussion

We compared actigraphic sleep and 24-hour RARs between cognitively unimpaired older adults with and without elevated Aβ deposition, measured at screening/baseline in a substudy of the A4 and LEARN studies. Contrary to our hypotheses, we found no significant differences between Aβ+ and Aβ− groups with regard to sleep parameters or standard cosinor or nonparametric RAR indices. When we applied a novel data-driven method, FOSR, to analyze RARs, however, we found that Aβ+ participants had lower mean activity levels than Aβ− participants from late in the night until the early morning (11:30 pm to 3:00 am), and higher activity levels in the early morning hours (4:30 am to 8:30 am) and from midday to late afternoon (12:30 pm to 5:30 pm). In addition, FOSR results revealed differences between groups in the variability of activity across days, with Aβ+ participants exhibiting more variable levels of activity across days than Aβ− participants from 9:30 pm to 1:00 am and from 4:30 am to 8:30 am, and greater stability of activity levels across days between 2:30 am and 3:30 am than Aβ− participants.

The lack of observed associations of Aβ deposition with actigraphic sleep and standard cosinor and nonparametric RAR parameters conflicts with prior investigations that have employed actigraphy. These include two studies in a different cohort by Ju et al., in which actigraphic sleep efficiency and WASO differed by Aβ status [6], and by Musiek et al., in which Aβ deposition was associated with greater within-day fragmentation (IV), greater across-day stability (IS), and greater cosinor amplitude [8]. However, our discrepant findings may be due to the relatively limited statistical power afforded by our sample size. Indeed, the two prior studies had larger samples (*N* = 142 in both) than the present one. On the other hand, when we used FOSR models, we observed associations of Aβ deposition with both the timing of activity and the variability of that activity across days. Thus, FOSR and other data-driven metrics with high temporal resolution may be able to detect differences in 24-hour RARs between cognitively unimpaired individuals with and without Aβ deposition to which conventional nonparametric RAR indices may not be as sensitive. Indeed, in a larger sample, Musiek et al. reported a marginally higher RAR amplitude (computed using the cosinor model) in Aβ+ participants than in Aβ− participants [8]. We did not detect a significant difference in that amplitude metric; however, when using FOSR, we found significantly higher mean activity among Aβ+ participants compared to Aβ− participants in two temporal windows, and lower activity among Aβ+ participants in one window. This may have manifested in a significant difference in amplitude if we had a larger sample. It is also possible that the use of different PET tracers in this study (florbetapir) and the Musiek et al. article (PiB) may account for differences in results. Moreover, that study found significant links of fragmented rhythms with AD pathology as measured by the ratio of phosphorylated tau to Aβ42 in CSF [8]. Thus, tau may be an important contributor to RAR fragmentation and warrants further investigation. Unfortunately, tau measurements are not available for the majority of participants in the actigraphy study, precluding their inclusion in this analysis.

The post-hoc finding that preclinical AD was associated with greater activity in the early morning and afternoon is, to our knowledge, novel. These changes may reflect alterations in the circadian timing system in preclinical AD [8], which would parallel the evidence for disrupted RARs in symptomatic AD [9]. The latter may be due to loss of volume in the suprachiasmatic nucleus (SCN) [26], and of particular cell populations (e.g. vasoactive intestinal peptide-expressing neurons) within the SCN [27]. Unfortunately, we cannot determine whether this elevated activity reflects goal-directed behavior (e.g. greater engagement in daily activities, exercise for fitness) or non-goal-directed behavior that might be termed “restlessness.” If it is the latter, actigraphy may be identifying a signal of restlessness associated with amyloid positivity, which might manifest in neuropsychiatric symptoms such as agitation, irritability, disinhibition, or aberrant motor behavior as AD progresses. However, without detailed neuropsychiatric symptom assessment, we cannot confirm this in this cohort. There is growing evidence for neuropsychiatric symptoms in cognitively intact older adults being risk factors for cognitive and functional decline [28, 29] and this actigraphic finding may reflect a similar association.

With respect to within-subject variability in 24-hour RARs across days, we found no difference in the nonparametric IS metric between Aβ+ and Aβ− participants, contrary to the findings of Musiek et al. [8] However, FOSR analyses revealed that variability across days in the level of activity at particular times of day—measured by the SD of each participant’s activity at a given time—differed between Aβ+ and Aβ− participants. Compared to their Aβ− counterparts, Aβ+ participants exhibited greater variability during two windows of the 24-hour day that, when combined totaled 7.5 h; they only showed lower variability in a single 1-hour window. As with the aforementioned finding of differences in mean activity levels at particular times of day, these results may reflect a deterioration of circadian timing mechanisms. The choice of temporal resolution is also something that needs to be explored in future work. As has been shown previously, the results of analyses using nonparametric RAR metrics may be sensitive to the choice of the time resolution [30, 31].

The FOSR methods used in this paper provide a powerful analytical tool to model time-of-day information and day-to-day variability in 24-hour RAR profiles. Functional regression borrows information across neighboring time points of the functional outcome and hence is more powerful in detecting interpretable temporally local differences compared to the conventional univariate approaches. Functional data approaches have demonstrated their high sensitivity in detecting associations in studies of cognition in older adults [32] and populations with mood disorders [33, 34].

However, a clear word of caution regarding our FOSR-derived results is needed. The nominally significant FOSR findings we have highlighted based on “simultaneous confidence intervals” are from analyses that accounted for the correlation of neighboring time points, and were, therefore, more conservative than approaches that ignore this feature of the data when evaluating statistical significance. Nonetheless, FOSR is a relatively new method and there are not yet widely accepted standards for establishing significance (i.e. statistical inference) with this method of high-volume data analysis. Thus, whether our analyses were sufficiently conservative is a question that is not easily answered. Indeed, when we used global *F*-tests to determine whether the mean level of activity or its variability differed between groups, results were nonsignificant; however, the *F*-test assesses effects across an entire 24-hour period, and may therefore be too conservative to detect differences that could be of theoretical or practical importance but are confined to, say, only 3 h of the 24-hour day. Finally, FOSR does not account for the circular nature of the 24-hour day and does not consider temporal adjacency of the beginning of the daily cycle (the time of the day right after 12 am) and the very end of the daily cycle (the time of the day right before 12 am). This likely results in a loss of power and should be considered in future methods-development work.

The present study has several limitations that must be considered when interpreting our results. First, all participants were non-Hispanic White and had high educational attainment. Future studies in more diverse samples will be important to evaluate the generalizability of our findings. Also, as discussed above, our sample size may have impaired our ability to detect true differences in sleep and RAR parameters, and the application of FOSR to studies of RARs and neuroimaging biomarkers is only a recent development for which consensus methodological guidelines are lacking. In addition, the present study focused on Aβ deposition and did not include any other important AD biomarkers, such as measures of tau from CSF or PET. The lack of a tau biomarker precluded us from investigating how sleep and RARs differ across the “A/T/N” framework, in which individuals are categorized based on being positive or negative for a biomarker for Aβ (A), tau (T), and neurodegeneration (N) [35]. Whether FOSR can be used to identify differences in RARs between those with and without preclinical AD-related neurodegeneration, or for that matter, with and without biomarkers specific to other neurodegenerative diseases (e.g. synucleinopathies) remains to be seen. Nonetheless, our results suggest this data-driven approach to the analysis of actigraphy data may be more sensitive to subtle signals in actigraphy data than the conventional actigraphic methods that they complement. This could be of particular value for discovery-oriented science, but the greater consensus is needed on how to establish statistical significance when employing these novel methods.

Additional limitations include the lack of data required to examine links of sleep and RARs with neuropsychiatric symptoms. Further, the median interval between PET scans and actigraphy across the two study groups was 52 days, and descriptive statistics suggested that this interval differed between Aβ+ and Aβ− groups. Although this difference is unlikely to have meaningfully affected results, if any Aβ− participants converted to Aβ+ during this interval, this would result in misclassification that would likely bias results toward the null, such that our findings reflect an underestimate of true differences between the Aβ+ and Aβ− groups. Finally, the present study was cross-sectional in design. Prospective studies are needed to evaluate whether FOSR-derived RARs can predict the change in cognition or AD biomarkers in cognitively unimpaired older adults who are Aβ+ or Aβ−.

In sum, we found no differences between cognitively unimpaired older adults who were Aβ+ and those who were Aβ− when using conventional actigraphic sleep parameters or 24-hour RAR metrics. Use of a novel data-driven method called FOSR, however, revealed between-group differences, both in the average 24-hour activity profiles of Aβ+ and Aβ− participants, and in their variability. FOSR may uncover differences between individuals with and without preclinical AD to which standard actigraphic analyses are less sensitive, and should be examined further as a potential marker of Aβ deposition. In addition, future studies are needed investigating the extent to which the magnitude of difference during the specific daily intervals identified here predict subsequent cognitive and functional trajectories in persons with Aβ deposition, and whether these 24-hour rhythm features predict Aβ deposition when observed in Aβ− persons. Results to this effect may point to potential therapeutic targets for circadian therapies to prevent or slow these outcomes.

## Supplementary Material

zpab015_suppl_Supplementary_Figure_S1-S2

zpab015_suppl_Supplementary_Figure_S3

zpab015_suppl_Supplementary_Figure_S4

zpab015_suppl_Supplementary_Figure_S5

zpab015_suppl_Supplementary_File

## Data Availability

Pre-randomization data from A4 are available here: https://ida.loni.usc.edu/login.jsp. Please contact the first author (Spira) with requests for actigraphy data.
