# Supplementary figures and images for: Brain amyloid burden, sleep, and 24-hour rest/activity rhythms: screening findings from the Anti-Amyloid Treatment in Asymptomatic Alzheimer’s and Longitudinal Evaluation of Amyloid Risk and Neurodegeneration Studies
Source: Sleep Adv. 2021 Sep 19;2(1):zpab015. doi: 10.1093/sleepadvances/zpab015 (PMC8519157; doi:10.1093/sleepadvances/zpab015)

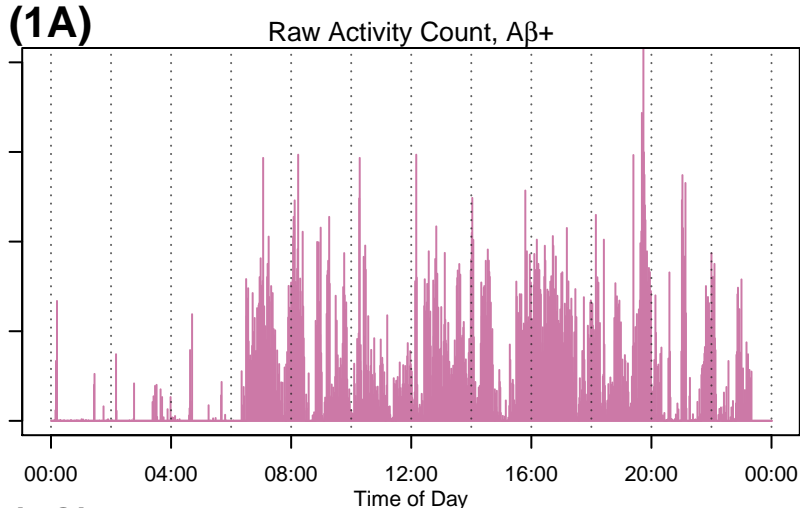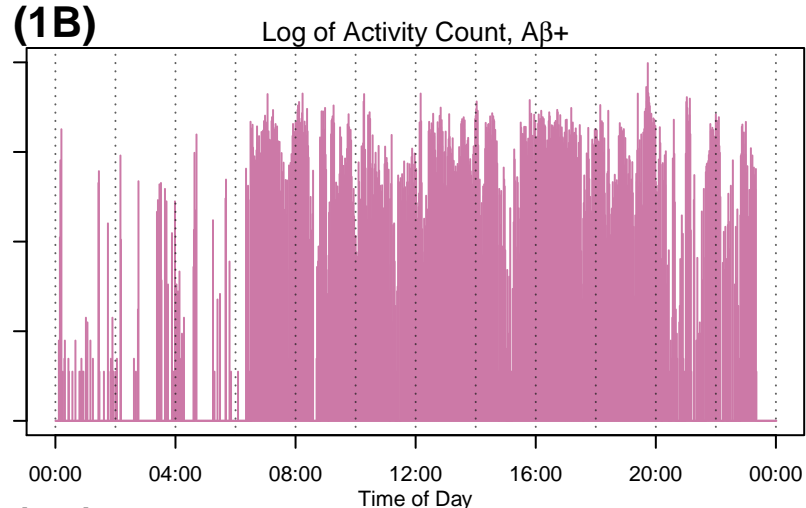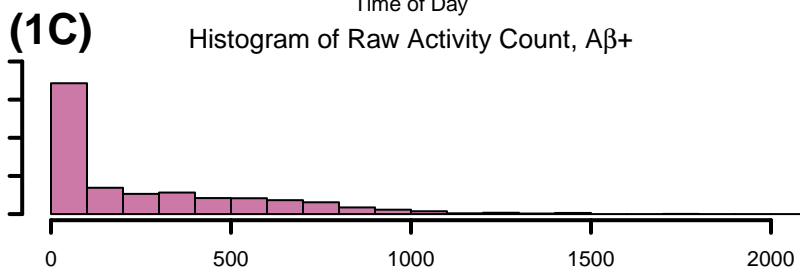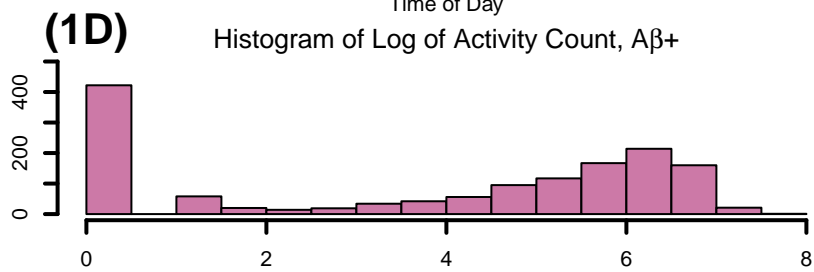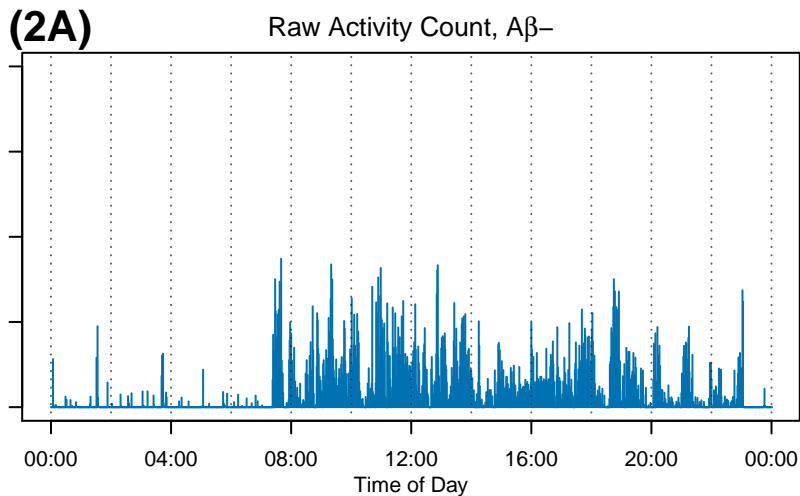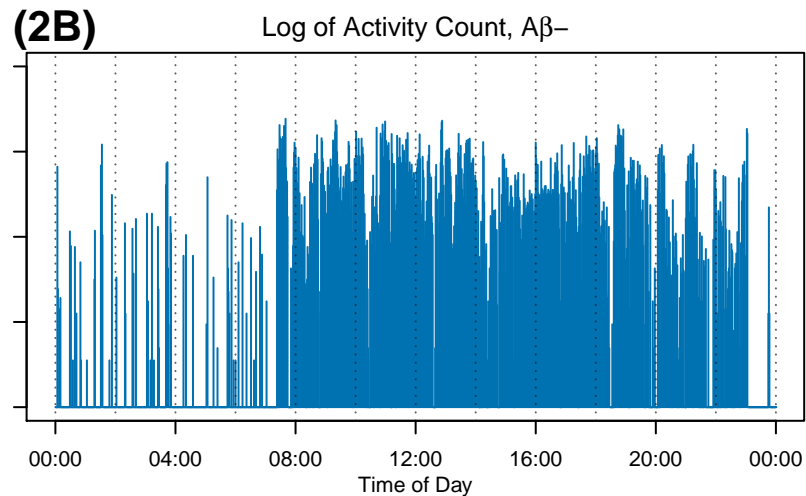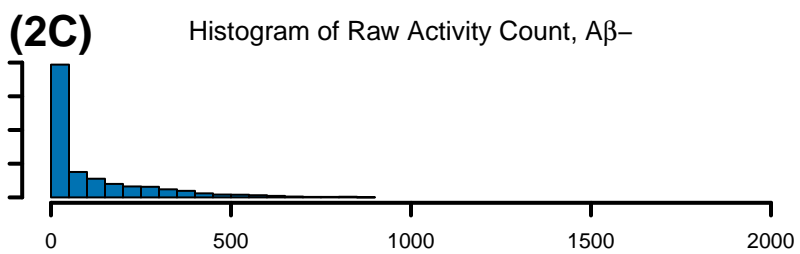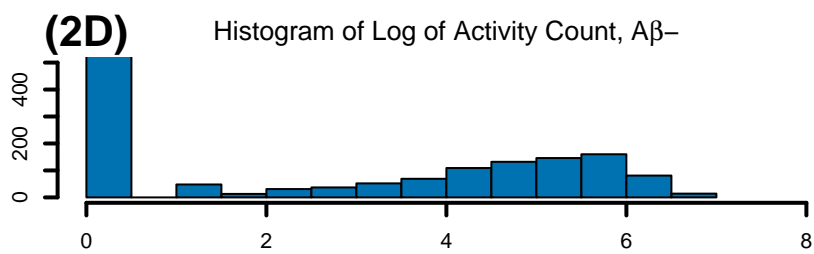

Supplement: zpab015_suppl_Supplementary_Figure_S1-S2 [file zpab015_suppl_supplementary_figure_s1-s2.pdf]

**(3A)** Average of Log of Activity Count (30-min),  $A\beta^+$

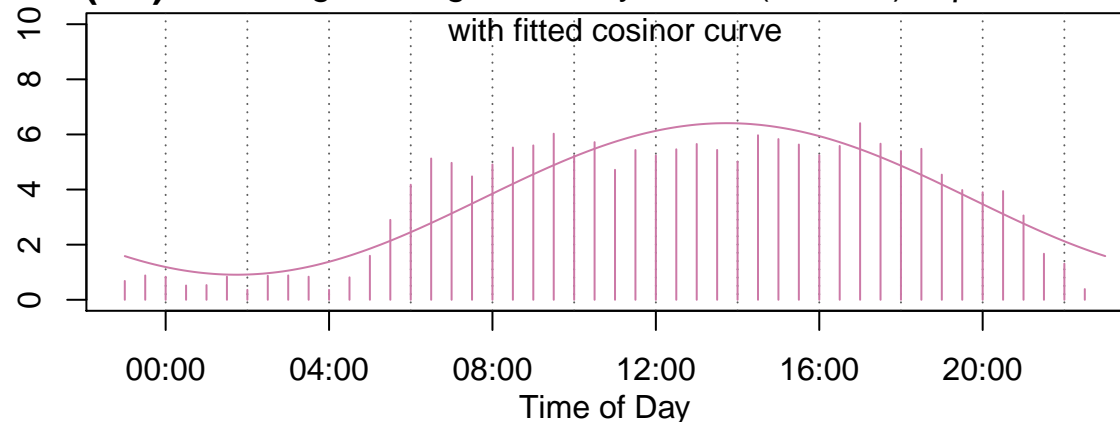

**(3B)** Average of Log of Activity Count (30-min),  $A\beta^-$

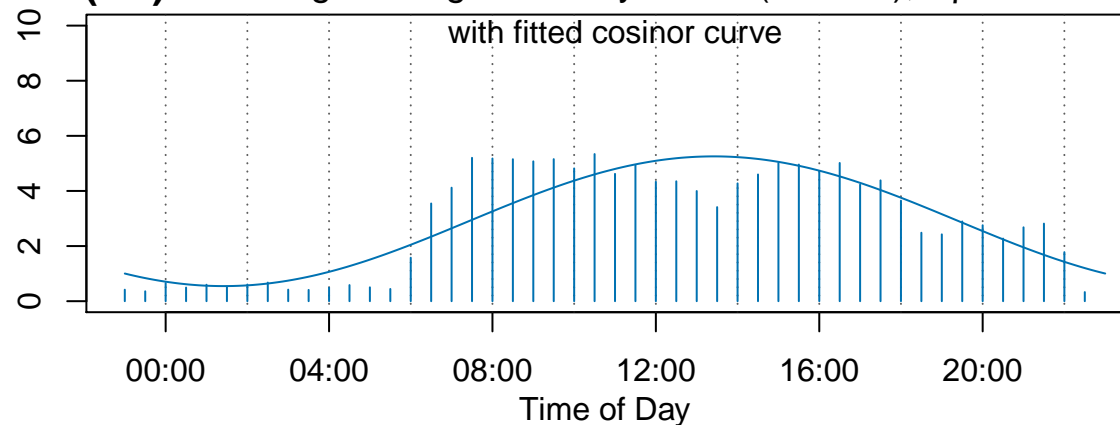

Supplement: zpab015_suppl_Supplementary_Figure_S3 [file zpab015_suppl_supplementary_figure_s3.pdf]

Average of Log of Activity Counts  
(60-minute intervals)

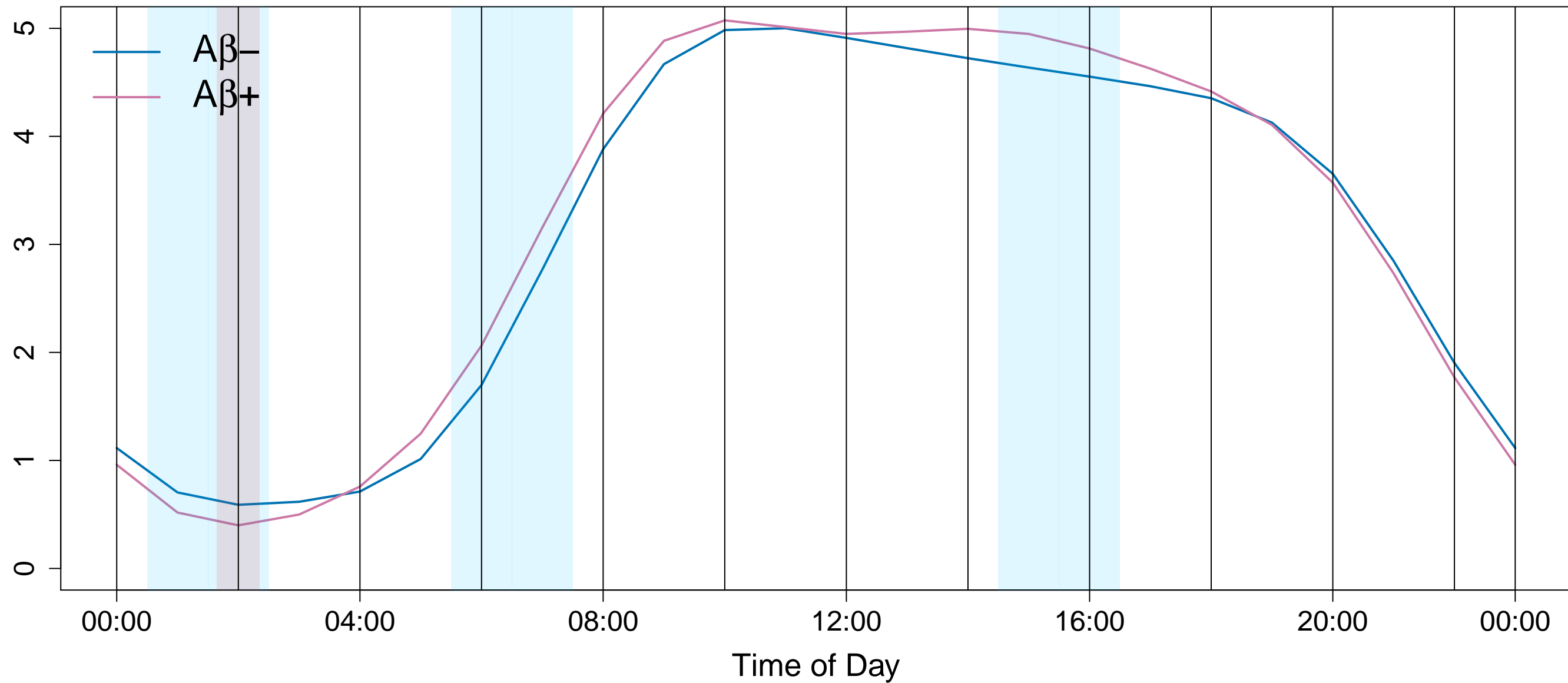

Supplement: zpab015_suppl_Supplementary_Figure_S4 [file zpab015_suppl_supplementary_figure_s4.pdf]

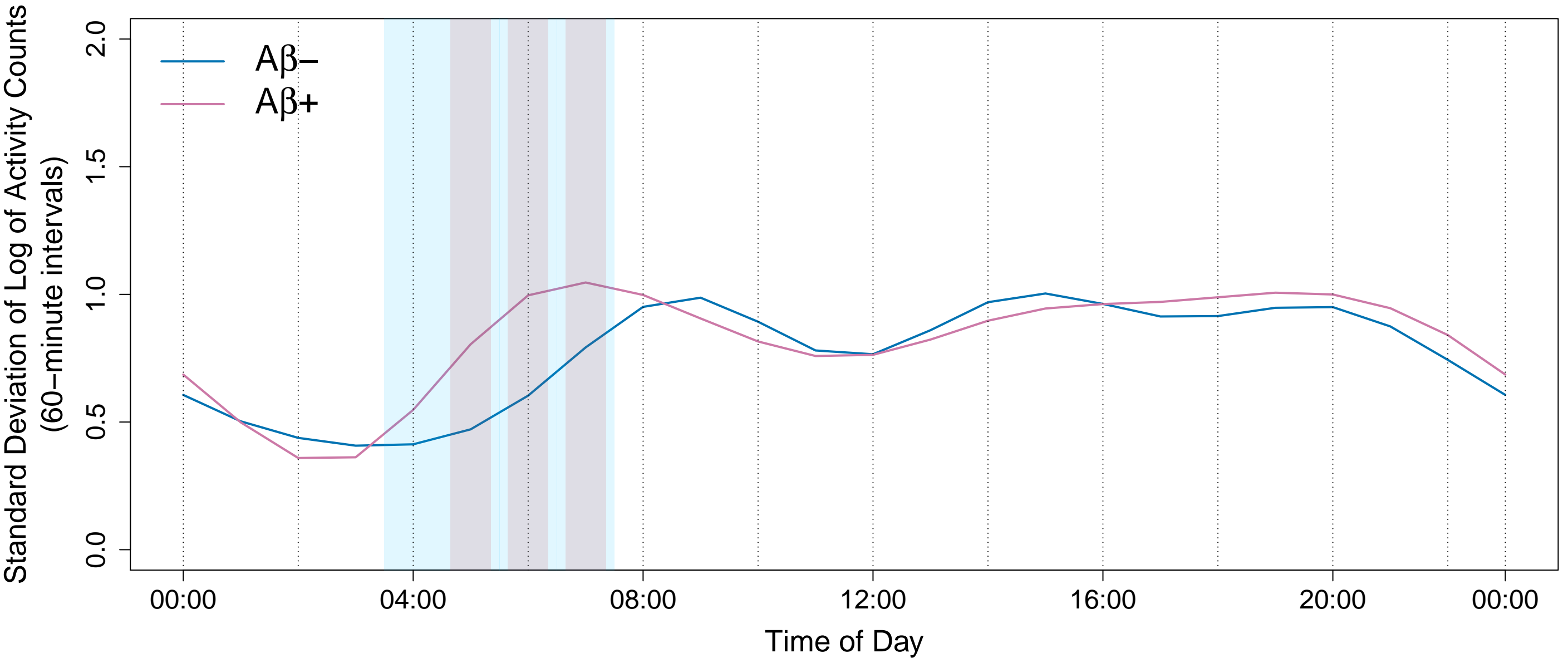

Supplement: zpab015_suppl_Supplementary_Figure_S5 [file zpab015_suppl_supplementary_figure_s5.pdf]
