# Supplementary material for: Brain amyloid burden, sleep, and 24-hour rest/activity rhythms: screening findings from the Anti-Amyloid Treatment in Asymptomatic Alzheimer’s and Longitudinal Evaluation of Amyloid Risk and Neurodegeneration Studies
Source: Sleep Adv. 2021 Sep 19;2(1):zpab015. doi: 10.1093/sleepadvances/zpab015 (PMC8519157; doi:10.1093/sleepadvances/zpab015)
Supplement: zpab015_suppl_Supplementary_File [file zpab015_suppl_supplementary_file.docx]

Brain Amyloid Burden, Sleep, and 24-Hour Rest/Activity Rhythms:

Screening Findings from A4 and LEARN

Adam P. Spira,^a,b,c^ Vadim Zipunnikov,^a^ Rema Raman,^d^ Jiyoon Choi,^d^ Junrui Di,^a^ Jiawei Bai, ^a^ Cynthia M. Carlsson,^e^ Jacobo E. Mintzer,^f^ Gad A. Marshall,^g^ Anton P. Porsteinsson,^h^ Roy Yaari,^i^ Sarah K. Wanigatunga,^a^ John Kim,^b^ Mark N. Wu,^b^ Paul S Aisen,^d^ Reisa A. Sperling,^g^

& Paul B. Rosenberg^b^

^a^Johns Hopkins Bloomberg School of Public Health, 624 N. Broadway, Hampton House, Rm. 794, Baltimore, MD, USA

^b^Johns Hopkins University School of Medicine, Baltimore, MD, USA

^c^Johns Hopkins Center on Aging and Health, Baltimore, MD, USA

^d^Alzheimer's Therapeutic Research Institute, University of Southern California, San Diego, CA, USA

^e^Wisconsin Alzheimer's Disease Research Center, University of Wisconsin School of Medicine and Public Health, Madison, WI, USA,

^f^Ralph H. Johnson VA Medical Center, Charleston, SC, USA and Lowcountry Center for Veterans Research, South Carolina Institute for Brain Health, Charleston, SC, USA

^g^Center for Alzheimer Research and Treatment, Brigham and Women’s Hospital, Massachusetts General Hospital, Harvard Medical School, Boston, MA, USA

^h^University of Rochester School of Medicine and Dentistry, Rochester, NY, USA

^i^Eli Lilly and Company

Correspondence: Adam P. Spira, Ph.D., 624 N. Broadway, Hampton House, Rm. 794, Baltimore, MD, 21205, USA. Email: [aspira@jhu.edu](mailto:aspira@jhu.edu). Tel: (410) 614-9498. Fax (410) 614-7469.

Supplemental Figures 1 and 2. Raw activity counts, log-transformed activity counts, and their distributions for an Aβ+ participant (Figure 1A-1D) and an Aβ- participant (Figures 2A-2D) .

Supplemental Figure 3. Average RAR profiles and fitted standard one-component cosinor models for an Aβ+ participant (Figure 3A) and an Aβ- participant (Figure 3B).

Supplemental Figure 4. Between-group differences in mean activity profiles (60-minute intervals). In analyses applying simultaneous confidence intervals, compared to Aβ- (LEARN) participants, Aβ+ (A4) participants had lower mean activity (mean log activity counts) between 2:00 AM and 3:00 AM only (pink bar identifies interval with significant differences). Additional differences were evident when we used the less conservative pointwise method of statistical inference (blue bars).

Supplemental Figure 5. Between-group differences in variability (standard deviation) of activity profiles (60-minute intervals). In analyses applying simultaneous confidence intervals, Aβ+ (A4) participants had greater variability in their activity between 5:00 AM and 8:00 AM (pink bars identify intervals with significant differences). Differences over a wider temporal interval emerged when we applied pointwise confidence intervals (blue bars).

Supplemental Table 1. Standard 24-hour rest/activity rhythm (RAR) indices (60-minute intervals), mean ±SD

|  | All participants | Aβ+ (A4) | Aβ- (LEARN) | *p*-value |
| --- | --- | --- | --- | --- |
|  | *N* = 57 | *n* = 26 | *n* = 31 |  |
| Cosinor indices |  |  |  |  |
| Amplitude | 2.30 ±0.35 | 2.33 ±0.33 | 2.27 ±0.37 | 0.509 |
| Mesor | 3.16 ±0.32 | 3.20 ±0.28 | 3.12 ±0.35 | 0.354 |
| Acrophase | 14:58PM ±1:0 | 14:47PM ±0:5 | 15:08PM±1:15 | 0.193 |
| Non-parametric indices |  |  |  |  |
| Relative amplitude (RA) | 0.80 ±0.08 | 0.81 ±0.07 | 0.80 ±0.08 | 0.879 |
| Interdaily stability (IS) | 0.81 ±0.07 | 0.80 ±0.06 | 0.81 ±0.07 | 0.368 |
| Intradaily variability (IV) | 0.48 ±0.13 | 0.45 ±0.11 | 0.50 ±0.15 | 0.172 |

Supplemental Table 2. Adjusted* associations of Aβ status with standard 24-hour rest/activity rhythm (RAR) indices (60-minute intervals)

|  | B (Standard Error)  *p*-value | | | | | | |
| --- | --- | --- | --- | --- | --- | --- | --- |
|  | Cosinor indices | | |  | Non-parametric indices | | |
|  | Amplitude | Mesor | Acrophase |  | Relative amplitude (RA) | Interdaily stability (IS) | Intradaily variability (IV) |
| Aβ+ (A4)  *n* = 26 | 0.07 (0.09)  *p* = 0.461 | 0.11 (0.09)  *p* = 0.229 | -0.35 (0.30)  *p* = 0.247 |  | 0.01 (0.02)  *p* = 0.820 | -0.02 (0.02)  *p* = 0.246 | -0.05 (0.04)  *p* = 0.163 |
| Aβ- (LEARN)  *n* = 31 | (ref.) | (ref.) | (ref.) |  | (ref.) | (ref.) | (ref.) |

*Adjusted for age, sex, education.
